# Quantitative Radiographic Morphology of Posterior Calcaneal Spurs Independently Predicts Patient-Centered Outcomes After Extracorporeal Shockwave Therapy for Insertional Achilles Tendinopathy: An MCID and PASS Analysis

**DOI:** 10.3390/jcm15041538

**Published:** 2026-02-15

**Authors:** Bilal Aykaç, Mustafa Dinç, Hünkar Çağdaş Bayrak, Recep Karasu

**Affiliations:** 1 Orthopedics and Traumatology Clinics, Bursa City Hospital, Bursa 16110, Turkey; drindianster@gmail.com (M.D.); recepkarasu@hotmail.com (R.K.); 2 Orthopedics and Traumatology Clinics, Çekirge State Hospital, Bursa 16090, Turkey; cagdasbayrak90@gmail.com

**Keywords:** achilles tendinopathy, extracorporeal shockwave therapy, calcaneal spur, minimal clinically important difference, patient acceptable symptom state

## Abstract

**Background/Objectives**: Insertional Achilles tendinopathy (IAT) is frequently associated with posterior calcaneal spurs; however, the prognostic significance of spur morphology for patient-centered treatment outcomes remains unquantified. This study aimed to establish treatment-specific minimal clinically important difference (MCID) and patient acceptable symptom state (PASS) thresholds after extracorporeal shockwave therapy (ESWT) and to determine whether quantitative spur morphology independently predicts achievement of these patient-centered endpoints. **Methods**: In this retrospective cohort study, 201 patients with IAT and radiographically confirmed posterior calcaneal spurs received standardized ESWT (three weekly sessions, 0.20 mJ/mm^2^, 8 Hz). Spur length and angle were measured on calibrated weight-bearing lateral radiographs. MCID and PASS thresholds for VISA-A, AOFAS, and VAS scores were determined using anchor-based receiver operating characteristic (ROC) analyses. Optimal spur morphology thresholds were derived from ROC curves using PASS achievement as the outcome criterion and the Youden index for cut-off selection. Multivariable logistic regression analyses, adjusted for age, sex, and body mass index, were performed to assess the independent prognostic value of spur morphology. **Results**: MCID thresholds were: ΔVISA-A ≥ 16.5 (AUC = 0.886), ΔAOFAS ≥ 11.5 (AUC = 0.830), and ΔVAS ≥ 2.5 (AUC = 0.897). PASS thresholds were: VISA-A ≥ 70.5 (AUC = 0.712), AOFAS ≥ 72.5 (AUC = 0.842), and VAS ≤ 3.5 (AUC = 0.753). While significant mean improvements occurred (all *p* < 0.001), only 36.8–43.3% of patients achieved MCID and 38.3–53.2% achieved PASS. ROC analysis identified spur length > 8.7 mm (AUC = 0.713) and spur angle > 16° (AUC = 0.738) as optimal thresholds predictive of PASS failure. In multivariable analysis, increased spur length (adjusted OR = 0.23–0.24, *p* < 0.001) and angle (adjusted OR = 0.16–0.23, *p* < 0.001) independently reduced the likelihood of achieving both MCID and PASS. **Conclusions**: This study provides the first anchor-based MCID and PASS thresholds for ESWT in IAT and demonstrates that posterior calcaneal spur morphology—specifically length > 8.7 mm and angle > 16°—independently predicts patient-defined treatment success. These findings support the integration of quantitative spur assessment into clinical decision-making for personalized management of IAT.

## 1. Introduction

Insertional Achilles tendinopathy (IAT) poses a distinct therapeutic challenge, not only because of its recalcitrant pain and functional impairment in active individuals but also due to the frequent coexistence of posterior calcaneal spurs, whose clinical significance remains controversial [1,2,3]. Unlike mid-portion Achilles tendinopathy, IAT involves a complex pathological interaction between the Achilles tendon, retrocalcaneal bursa, and posterosuperior calcaneal bone, often accompanied by spur formation at the tendon–bone interface [4,5,6]. Although these osseous prominences are readily identifiable on lateral radiographs, their precise role in symptom persistence and treatment response remains incompletely understood.

Extracorporeal shockwave therapy (ESWT) has emerged as a cornerstone nonoperative treatment for chronic Achilles tendinopathy, supported by evidence demonstrating its analgesic, anti-inflammatory, and tissue-regenerative effects [7,8,9,10]. Proposed biological mechanisms include stimulation of neovascularization, modulation of nociceptive pathways, upregulation of growth factors, and enhanced tenocyte activity at the tendon–bone junction [11,12,13,14]. Despite these favorable mechanisms and encouraging clinical results, treatment response to ESWT remains heterogeneous, with a substantial proportion of patients failing to achieve satisfactory symptomatic or functional outcomes [15,16,17]. This variability underscores the critical need to identify robust modulators of treatment efficacy, which likely include not only patient-related factors but also distinct structural characteristics of the insertional pathology.

Radiographic calcaneal spurs represent a hallmark finding in insertional Achilles tendinopathy; however, previous studies have largely focused on their presence or absence rather than their detailed morphological features such as angle and length [18,19,20,21]. Emerging biomechanical and imaging-based evidence suggests that spur geometry—particularly angular orientation and length—may substantially alter local stress distribution at the Achilles enthesis, perpetuating repetitive microtrauma and potentially counteracting the biological reparative response induced by ESWT [2,5,22,23]. Nevertheless, despite this plausible mechanistic rationale, the prognostic value of quantitative calcaneal spur morphology in predicting clinical outcomes following ESWT has not been systematically investigated.

Another important limitation in the current literature is the reliance on statistical significance alone when interpreting treatment success. Improvements in patient-reported outcome measures (PROMs) do not necessarily correspond to changes that patients perceive as meaningful or satisfactory in daily life [24,25,26,27]. The minimal clinically important difference (MCID) represents the smallest change in an outcome measure that patients perceive as clinically meaningful, whereas the patient acceptable symptom state (PASS) defines the threshold beyond which patients consider their symptoms acceptable. To address this gap, the concepts of MCID and PASS have gained prominence as clinically interpretable benchmarks that link numerical score changes to patient-perceived benefit [28,29]. Although MCID and PASS thresholds have been defined for several musculoskeletal disorders, data specific to ESWT-treated insertional Achilles tendinopathy remain scarce. Critically, no previous study has integrated anchor-based MCID and PASS analyses with quantitative radiographic assessment of calcaneal spur morphology to evaluate predictors of clinically meaningful improvement following ESWT. Identifying whether specific morphological thresholds of posterior calcaneal spurs are associated with a reduced likelihood of achieving a patient-acceptable outcome could provide valuable guidance for patient selection, expectation management, and individualized treatment strategies.

Therefore, the primary aim of this study was to determine MCID and PASS thresholds for commonly used clinical outcome measures in patients with insertional Achilles tendinopathy treated with ESWT. The secondary aim was to investigate whether posterior calcaneal spur morphology—specifically spur angle and spur length—predicts the probability of achieving a satisfactory clinical state. We hypothesized that (1) valid MCID and PASS thresholds would be established for this patient population, and (2) a more horizontally oriented (greater angular prominence) and longer calcaneal spur would act as independent negative predictors for attaining PASS following ESWT.

## 2. Materials and Methods

### 2.1. Study Design and Ethical Approval

This study was designed as a retrospective observational cohort study evaluating clinical outcomes and prognostic factors in patients with insertional Achilles tendinopathy (IAT) treated with extracorporeal shockwave therapy (ESWT). The study was conducted at a single tertiary referral orthopedic center. Ethical approval was obtained from the institutional review board (approval no: 2025-14/15, date: 6 August 2025) prior to data collection, and all procedures were performed in accordance with the principles of the Declaration of Helsinki.

### 2.2. Patient Selection and Eligibility Criteria

Consecutive patients who underwent extracorporeal shockwave therapy (ESWT) for insertional Achilles tendinopathy between January 2021 and January 2025 were screened for eligibility. During this period, 260 patients were identified. Inclusion criteria were: (1) age ≥ 18 years; (2) posterior heel pain localized to the Achilles tendon insertion persisting for at least 3 months; (3) clinical findings consistent with insertional Achilles tendinopathy, including insertional tenderness and activity-related pain; (4) radiographic confirmation of insertional pathology with the presence of a posterior calcaneal spur on standardized weight-bearing lateral ankle radiographs; and (5) completion of a minimum 6-month follow-up.

Patients were excluded if they had a history of Achilles tendon surgery, acute Achilles tendon rupture, systemic inflammatory or rheumatologic disease, neurological disorders affecting lower limb function, corticosteroid injection to the Achilles region within the preceding 6 months, concomitant foot or ankle pathology that could confound outcome assessment (including symptomatic Haglund’s deformity), or incomplete clinical or radiographic data. After applying the inclusion and exclusion criteria, 201 patients were included in the final analysis. These criteria were applied to minimize confounding factors affecting tendon healing and patient-reported outcomes, while specifically enabling isolation of the prognostic effect of posterior calcaneal spur morphology.

### 2.3. Extracorporeal Shockwave Therapy Protocol

All patients were treated using a standardized extracorporeal shockwave therapy protocol with an electromagnetic shockwave device (DolorClast^®^ Smart 20, EMS Electro Medical Systems, Nyon, Switzerland), which has been extensively validated for the treatment of chronic Achilles tendinopathy [17,30,31]. All ESWT procedures were performed by the same experienced orthopedic surgeon to ensure procedural consistency. Each patient received three ESWT sessions at weekly intervals, consistent with widely adopted protocols for insertional Achilles tendinopathy [15,32]. During each session, 2000 shockwaves were delivered focally to the Achilles tendon insertion using palpation-guided localization [17]. No ultrasound or radiographic imaging was used for treatment localization or pre-procedural planning, and ESWT was applied exclusively using a palpation-guided approach targeting the point of maximal tenderness. Palpation-guided application was employed as it represents a commonly used and clinically practical approach in the management of insertional pathology, with existing studies indicating that it can yield clinical improvements similar to those achieved with image-guided methods [30,33]. Shockwaves were applied with an energy flux density of 0.20 mJ/mm^2^ and a frequency of 8 Hz [15,17]. These parameters were chosen to provide sufficient biological stimulation while maintaining patient tolerability and minimizing adverse effects [31,34]. Treatment intensity was gradually increased during the initial session according to patient tolerance. No local anesthesia or analgesic infiltration was used during ESWT administration. Previous experimental and clinical studies have demonstrated that local anesthesia may attenuate the therapeutic efficacy of ESWT by suppressing nociceptive-mediated signaling pathways that contribute to shockwave-induced tissue regeneration and neuromodulation [35,36,37]. Patients were advised to avoid nonsteroidal anti-inflammatory drugs throughout the treatment period and for one week following the final session to prevent interference with inflammatory processes essential for tendon healing [38,39].

### 2.4. Radiographic Assessment and Calibration

Standardized weight-bearing lateral ankle radiographs were obtained for all patients at baseline using a consistent imaging protocol to minimize positional variability. Prior to morphometric analysis, all digital radiographs were calibrated to ensure accurate linear measurements. Pixel-to-millimeter conversion was performed in ImageJ software version 1.53k (National Institutes of Health, Bethesda, MD, USA) using the embedded digital ruler provided by the radiographic imaging system, which applies a fixed and standardized scale to each image at the time of acquisition. This calibration procedure ensured accurate, reproducible, and comparable measurements of calcaneal spur length across all radiographs.

### 2.5. Calcaneal Spur Morphology Measurement

Posterior calcaneal spur morphology was evaluated quantitatively using ImageJ software (National Institutes of Health, Bethesda, MD, USA) by assessing spur length and spur angle on standardized weight-bearing lateral ankle radiographs. All radiographs were obtained with the patient in an upright position and the ankle in neutral dorsiflexion to minimize projectional variability and ensure consistency across measurements.

**Figure 1:**
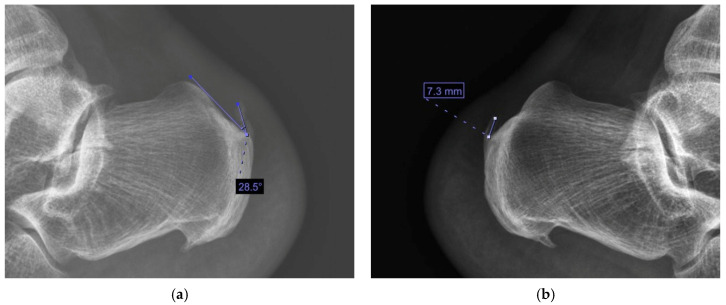
Radiographic measurement of posterior calcaneal spur morphology. (**a**) Spur length was defined as the maximal linear distance (in millimeters) measured from the posterior calcaneal cortex to the distal tip of the enthesophyte along the longitudinal axis of the spur. (**b**) Spur angle was defined as the angle formed between (i) the longitudinal axis of the enthesophyte and (ii) a reference line drawn tangential to the posterior calcaneal cortex at the base of the spur.

Spur length was defined as the maximal linear distance (in millimeters) from the posterior calcaneal cortex to the distal tip of the enthesophyte measured along its longitudinal axis. Spur angle was defined as the angle formed between the longitudinal axis of the spur and a reference line tangential to the posterior calcaneal cortex, reflecting the degree of horizontal prominence and potential mechanical impingement at the Achilles enthesis [2,23] (Figure 1). This geometric approach was selected to capture both the extent and orientation of spur prominence in a manner that is reproducible and directly applicable to routine clinical radiographs.

Because no standardized quantitative method currently exists for detailed assessment of posterior calcaneal spur geometry, this measurement strategy was designed to be simple, anatomically grounded, and clinically interpretable rather than theory-driven. Measurements were performed using fixed magnification and calibrated scale settings within ImageJ to ensure metric accuracy.

All measurements were independently performed by two observers blinded to clinical outcomes to ensure unbiased radiographic assessment. Each parameter was measured twice by each observer at separate time points, and the mean value was used for statistical analysis. Interobserver reliability was assessed using intraclass correlation coefficients (ICC), with ICC values greater than 0.80 considered indicative of excellent reliability.

### 2.6. Clinical Outcome Measures

Clinical outcomes were assessed at baseline and at 6-month follow-up using validated patient-reported outcome measures. Achilles tendon–specific function was evaluated using the Victorian Institute of Sports Assessment–Achilles questionnaire (VISA-A). Overall ankle and hindfoot function was assessed using the American Orthopaedic Foot and Ankle Society (AOFAS) Ankle–Hindfoot Score. Pain intensity was quantified using a 10-point visual analog scale (VAS). Patient-perceived change was assessed at follow-up using a 5-point Likert-type Global Rating of Change (GROC) scale, with response options ranging from 1 (“much worse”), 2 (“worse”), 3 (“no change”), 4 (“moderately better”), to 5 (“much better”).

### 2.7. Determination of MCID and PASS

Minimal clinically important difference (MCID) thresholds were determined using an anchor-based approach, with the Likert-type GROC scale serving as the external criterion. Because MCID aims to capture the smallest patient-perceived improvement, only patients reporting a positive change (GROC >3) were considered to have achieved a meaningful benefit. This corresponds to scores of ≥4 (“moderately better” or “much better”), thereby excluding responses of “no change” (score = 3) or worsening (scores 1–2). This threshold selection aligns with the conceptual definition of a minimal clinically important difference and is consistent with established anchor-based methodology in musculoskeletal research [25,26]. Receiver operating characteristic (ROC) curve analysis was used to identify optimal MCID cut-off values for each outcome measure based on the maximal Youden index. Area under the curve (AUC), sensitivity, specificity, and 95% confidence intervals were calculated to assess discriminatory performance.

Patient acceptable symptom state (PASS) was determined using the following standardized anchor question adapted from Tubach et al. [26]: “Considering your symptoms, pain, and functional limitations during the past month, do you consider your current condition acceptable?” Responses were recorded as “Yes” or “No.” Patients responding “Yes” were classified as having achieved PASS. ROC analysis using absolute outcome scores at final follow-up was employed to identify cut-off values representing an acceptable symptom state.

### 2.8. Statistical Analysis

All statistical analyses were performed using SPSS software (version 27.0; IBM Corp., Armonk, NY, USA). Continuous variables were summarized as mean ± standard deviation for normally distributed data or median (range) for non-normally distributed data, as appropriate. Categorical variables were presented as frequencies and percentages. Normality of continuous variables was assessed using the Shapiro–Wilk test.

Within-group changes in clinical outcome measures (VISA-A, AOFAS, and VAS) between baseline and 6-month follow-up were analyzed using paired *t*-tests for normally distributed variables and Wilcoxon signed-rank tests for non-normally distributed variables. Associations between changes in outcome scores and Global Rating of Change (GROC) responses were evaluated using Spearman’s rank correlation coefficient.

Minimal clinically important difference (MCID) thresholds were determined using an anchor-based approach, with the GROC scale serving as the external criterion. Receiver operating characteristic (ROC) curve analysis was performed for each outcome measure, and the optimal MCID cut-off values were identified based on the maximal Youden index (sensitivity + specificity − 1). Patient acceptable symptom state (PASS) thresholds were similarly determined using ROC analysis, with PASS achievement as the dependent outcome.

To evaluate the prognostic value of posterior calcaneal spur morphology, ROC analyses were conducted for spur angle and spur length with PASS achievement as the primary outcome. Optimal cut-off values for spur angle and spur length were identified using the maximal Youden index, selecting thresholds that best discriminated between patients who achieved and did not achieve PASS. Discriminatory performance was quantified using the area under the ROC curve (AUC) with 95% confidence intervals.

Patients were further stratified into four predefined spur morphology groups based on combinations of spur angle and spur length thresholds. Between-group differences in 6-month clinical outcomes were evaluated using the Kruskal–Wallis test due to non-normal distribution of outcome variables.

Multivariable logistic regression analysis was performed to determine whether posterior calcaneal spur morphology independently predicted achievement of MCID and PASS after ESWT. Variables entered into the regression models included age, sex, body mass index, symptom duration, baseline outcome scores, spur angle, and spur length. Results were expressed as odds ratios (ORs) with 95% confidence intervals.

Interobserver reliability for radiographic measurements was assessed using intraclass correlation coefficients (ICC), with ICC values greater than 0.80 considered indicative of excellent reliability. All statistical tests were two-tailed, and statistical significance was set at *p* < 0.05.

## 3. Results

### 3.1. Patient Characteristics

A total of 201 patients were included in the analysis, comprising 109 males (54.2%) and 92 females (45.8%), with a mean age of 45.08 ± 10.38 years and a mean body mass index (BMI) of 26.81 ± 3.52 kg/m^2^. Baseline symptom burden was moderate, with mean VISA-A score of 57.77 ± 13.13, VAS pain score of 6.27 ± 1.26, and AOFAS Ankle–Hindfoot Score of 61.75 ± 8.39. Posterior calcaneal spur morphology demonstrated substantial inter-individual variability. The mean spur angle was 16.28 ± 6.38° (range: 2.0–32.8°), and the mean spur length was 8.10 ± 2.94 mm (range: 1.8–16.1 mm) (Table 1).

### 3.2. Change in Clinical Outcomes After ESWT

At 6-month follow-up, patients demonstrated statistically significant improvements across all outcome measures. Mean VISA-A score increased from 57.77 ± 13.13 to 72.60 ± 14.89, VAS pain score decreased from 6.27 ± 1.26 to 4.04 ± 1.92, and AOFAS score increased from 61.75 ± 8.39 to 73.22 ± 11.49 (all *p* < 0.001). Corresponding mean change scores were ΔVISA-A = +14.83 ± 8.46, ΔVAS = −2.23 ± 1.54, and ΔAOFAS = +11.57 ± 8.48, indicating clinically relevant improvement at the cohort level (Table 2).

### 3.3. Anchor Validation: Association of GROC with Objective Change

To verify that the Global Rating of Change (GROC) scale reflected true clinical improvement, correlations between GROC scores and change values of each outcome measure were examined. GROC demonstrated a very strong positive correlation with improvement in VISA-A (r = 0.861, *p* < 0.001) and reduction in VAS pain (r = 0.848, *p* < 0.001). Improvement in AOFAS score also showed a strong correlation with GROC (r = 0.733, *p* < 0.001). These findings confirm the construct validity of GROC as an external anchor for clinically meaningful improvement in this cohort (Table 3).

### 3.4. MCID Thresholds and Responder Rates

**Figure 2:**
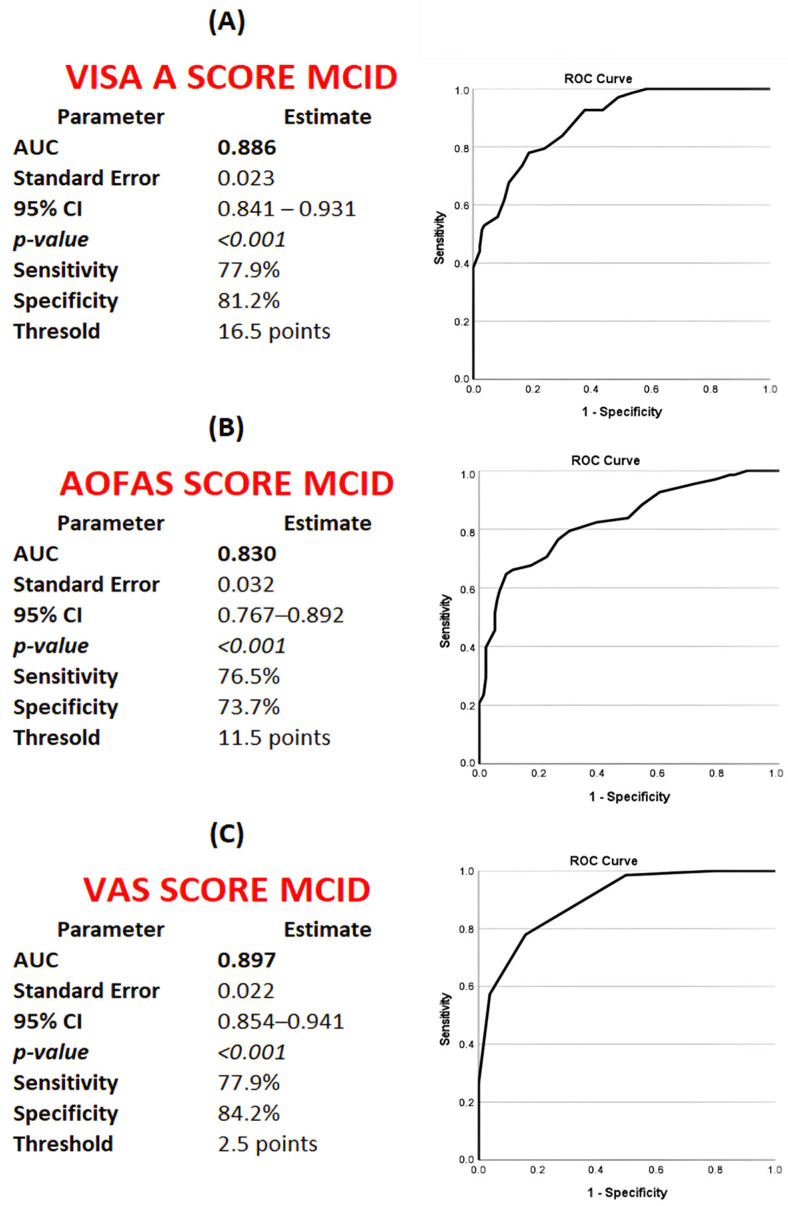
Receiver operating characteristic (ROC) curves for determination of minimal clinically important difference (MCID) thresholds for (**A**) VISA-A, (**B**) AOFAS Ankle–Hindfoot Score, and (**C**) VAS pain score following extracorporeal shockwave therapy in patients with insertional Achilles tendinopathy. MCID thresholds were derived using an anchor-based approach with the Global Rating of Change scale as the external criterion.

Receiver operating characteristic (ROC) analysis demonstrated excellent discriminatory performance for determining minimal clinically important difference (MCID) thresholds across all outcome measures. The optimal MCID cut-off values were identified as ΔVISA-A ≥ 16.5 (AUC = 0.886), ΔAOFAS ≥ 11.5 (AUC = 0.830), and VAS pain reduction ≥ 2.5 points (AUC = 0.897) (Figure 2). Based on these MCID thresholds, 78 patients (38.8%) achieved MCID for VISA-A, 87 patients (43.3%) for AOFAS, and 74 patients (36.8%) for VAS pain (Table 4).

At the optimal MCID cut-off values, ROC analyses also demonstrated clinically relevant predictive performance. VISA-A MCID achievement yielded a positive predictive value (PPV) of 73.4% and a negative predictive value (NPV) of 84.6%. Corresponding PPV and NPV values were 68.9% and 79.8% for AOFAS MCID, and 74.1% and 87.9% for VAS MCID, respectively (Table 5).

### 3.5. PASS Thresholds and Final-State Satisfaction

**Figure 3:**
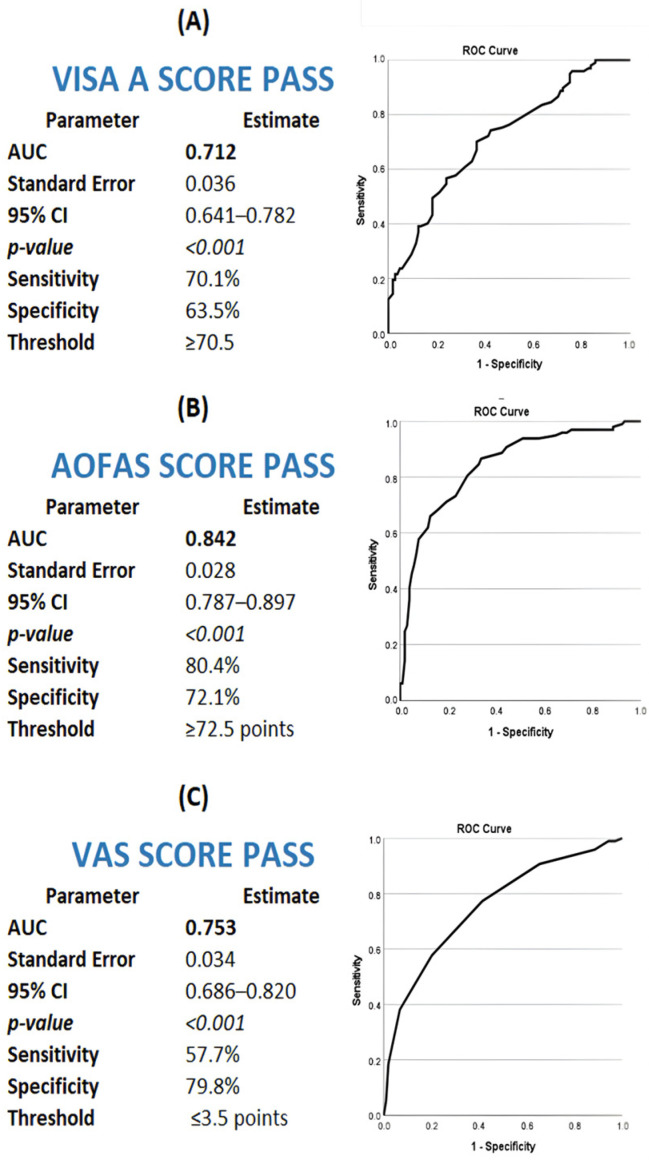
Receiver operating characteristic (ROC) curves for determination of patient acceptable symptom state (PASS) thresholds for (**A**) VISA-A, (**B**) AOFAS Ankle–Hindfoot Score, and (**C**) VAS pain score at 6-month follow-up after extracorporeal shockwave therapy in patients with insertional Achilles tendinopathy. PASS thresholds were derived using an anchor-based approach based on patient-reported satisfaction.

Patient acceptable symptom state (PASS) thresholds derived from final absolute outcome scores demonstrated variable discriminatory performance across instruments. Optimal PASS cut-offs were identified as final VISA-A ≥ 70.5 (AUC = 0.712), final AOFAS ≥ 72.5 (AUC = 0.842), and final VAS ≤ 3.5 (AUC = 0.753) (Figure 3). Using these thresholds, PASS was achieved by 106 patients (52.7%) according to VISA-A, 107 patients (53.2%) according to AOFAS, and 77 patients (38.3%) according to VAS pain (Table 6).

At the identified PASS cut-off values, ROC analyses demonstrated clinically meaningful predictive performance. AOFAS PASS showed the strongest discrimination, with a positive predictive value (PPV) of 76.1% and a negative predictive value (NPV) of 77.0%. VISA-A PASS demonstrated moderate predictive performance (PPV 69.2%, NPV 64.8%), whereas VAS PASS showed acceptable discrimination with a PPV of 65.3% and an NPV of 73.6% (Table 7).

### 3.6. Spur Morphology as a Predictor of PASS Achievement

**Figure 4:**
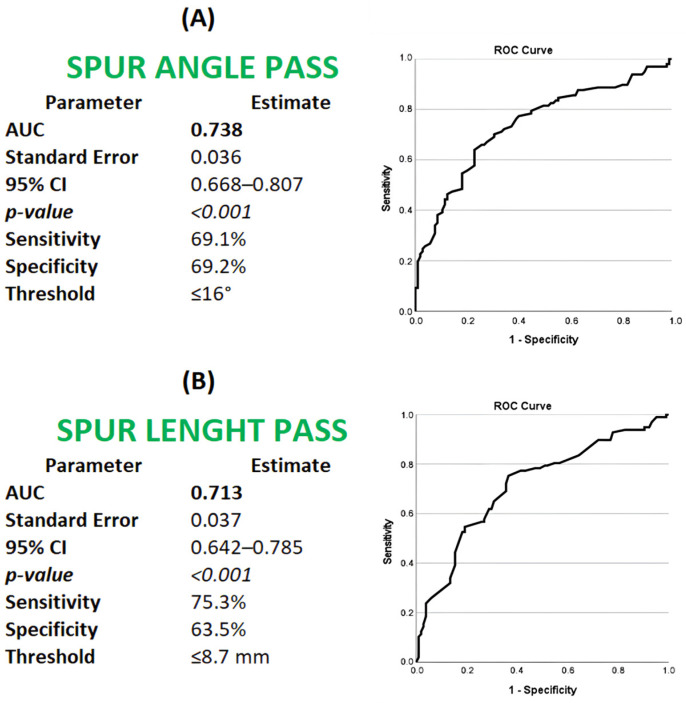
Receiver operating characteristic (ROC) curves illustrating the ability of posterior calcaneal spur morphology—(**A**) spur angle and (**B**) spur length—to predict achievement of the patient acceptable symptom state (PASS) at 6-month follow-up after extracorporeal shockwave therapy in patients with insertional Achilles tendinopathy.

Posterior calcaneal spur morphology demonstrated prognostic value for achieving a patient acceptable symptom state (PASS) at 6 months. Using PASS status (satisfied vs. not satisfied) as the binary outcome, receiver operating characteristic (ROC) analyses were performed for spur angle and spur length. Optimal cut-off values were selected based on the maximal Youden index (sensitivity + specificity − 1). This approach identified a spur angle of ≤16° (sensitivity 69.1%, specificity 69.2%, AUC 0.738; 95% CI 0.668–0.807) and a spur length of ≤8.7 mm (sensitivity 75.3%, specificity 63.5%, AUC 0.713; 95% CI 0.642–0.785) as the thresholds most discriminative for PASS achievement (Figure 4).

At the identified morphological thresholds, radiographic parameters demonstrated moderate predictive performance for PASS achievement. A spur angle of ≤16° yielded a positive predictive value (PPV) of 71.4% and a negative predictive value (NPV) of 66.6%, while a spur length of ≤8.7 mm demonstrated a PPV of 70.6% and an NPV of 68.5% (Table 8).

When stratified according to these ROC-derived thresholds, patients with spur angle ≤ 16° showed a higher PASS achievement rate than those with angle > 16° (59.6% vs. 46.1%), although the difference did not reach statistical significance (*p* = 0.075). In contrast, spur length was significantly associated with PASS: patients with spur length ≤ 8.7 mm achieved PASS more frequently than those with length > 8.7 mm (64.9% vs. 37.8%, *p* < 0.001) (Table 9).

### 3.7. Distribution of Final Outcomes According to Spur Morphology Groups

**Figure 5:**
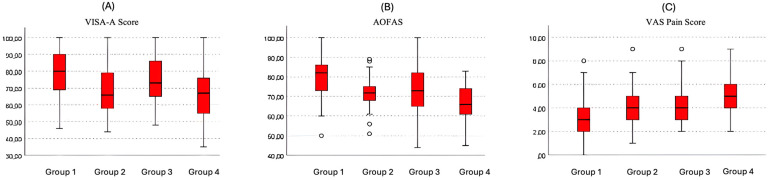
Six-Month Clinical Outcomes Across Posterior Calcaneal Spur Morphology Groups. Box-and-whisker plots illustrating 6-month clinical outcomes across four posterior calcaneal spur morphology groups stratified by spur angle (≤16° vs. >16°) and spur length (≤8.7 mm vs. >8.7 mm). The four groups represent the following combinations: (Group 1) angle ≤ 16° and length ≤ 8.7 mm, (Group 2) angle ≤ 16° and length > 8.7 mm, (Group 3) angle > 16° and length ≤ 8.7 mm, and (Group 4) angle > 16° and length > 8.7 mm. Panels show (**A**) VISA-A score, (**B**) AOFAS Ankle–Hindfoot Score, and (**C**) VAS pain score. Boxes represent median values and interquartile ranges; whiskers indicate values within 1.5× the interquartile range, and circles denote outliers. Between-group differences were assessed using the Kruskal–Wallis test.

When patients were stratified into four predefined spur morphology groups based on combined spur angle and spur length thresholds, significant between-group differences were observed in all 6-month clinical outcome measures (Figure 5).

Box-and-whisker plots demonstrated a graded deterioration in outcomes with increasing spur severity. Patients with favorable spur morphology (spur angle ≤ 16° and spur length ≤ 8.7 mm) exhibited the highest median VISA-A and AOFAS scores and the lowest median VAS pain scores at 6 months. In contrast, patients with unfavorable morphology (spur angle > 16° and spur length > 8.7 mm) showed progressively lower functional scores and higher pain levels.

Between-group comparisons using the Kruskal–Wallis test confirmed statistically significant differences across the four spur morphology groups for VISA-A, AOFAS, and VAS outcomes at 6 months (all *p* < 0.001), indicating a stepwise association between increasing posterior calcaneal spur severity and poorer clinical outcomes following extracorporeal shockwave therapy.

### 3.8. Multivariable Predictors of PASS and MCID Achievement

Multivariable logistic regression analysis demonstrated that both increased spur angle (>16°) and increased spur length (>8.7 mm) were independent negative predictors of achieving PASS and MCID after ESWT. After adjustment for age, sex, and BMI, spur angle >16° was associated with significantly lower odds of PASS achievement (adjusted OR = 0.23, *p* < 0.001) and MCID achievement (adjusted OR = 0.16, *p* < 0.001). Similarly, spur length >8.7 mm independently reduced the likelihood of PASS (adjusted OR = 0.23, *p* < 0.001) and MCID (adjusted OR = 0.24, *p* < 0.001). In contrast, demographic variables did not significantly influence outcomes (Table 10).

## 4. Discussion

This study provides two clinicaly relevant contributions to the contemporary management of insertional Achilles tendinopathy (IAT). First, it establishes the first anchor-based, treatment-specific minimal clinically important difference (MCID) and patient acceptable symptom state (PASS) thresholds for VISA-A, AOFAS, and VAS scores in an ESWT-treated IAT cohort. Second, and more innovatively, it demonstrates that quantitative radiographic characteristics of posterior calcaneal spur morphology—specifically spur angle and spur length—function as independent prognostic modifiers of patient-defined treatment success. Together, these findings bridge structural pathoanatomy with patient-centered outcomes and outline a potential framework for future research into individualized prognostic counseling.

A central finding of this study is the marked divergence between statistically significant group-level improvement and individual-level clinical success. Although mean improvements in VISA-A, AOFAS, and VAS scores were significant after ESWT, only 36.8–43.3% of patients achieved MCID, and 38.3–53.2% reached PASS, depending on the outcome measure. This discrepancy underscores the limitations of relying solely on mean score changes and highlights the clinical relevance of anchor-based thresholds, which translate numerical improvements into patient-perceived benefit.

The MCID thresholds identified (ΔVISA-A ≥ 16.5, ΔAOFAS ≥ 11.5, ΔVAS ≥ 2.5) represent the first anchor-based, treatment-specific estimates for ESWT in IAT. Previous ESWT studies have largely reported group-level outcomes or categorized success using ordinal satisfaction scales such as the Roles and Maudsley score [8,15,17,40,41,42,43], without defining quantitative thresholds for clinically meaningful individual improvement. Consequently, earlier literature could not distinguish between statistical improvement and patient-relevant change. By applying a Global Rating of Change anchor combined with ROC optimization, the present study addresses this methodological gap and enables direct clinical interpretation of treatment response [25,26].

PASS thresholds further extend this patient-centered framework by defining the absolute symptom state patients consider acceptable, conceptually distinct from improvement captured by MCID. This “feeling well” versus “feeling better” distinction is fundamental to outcome interpretation [26,44,45]. In this cohort, PASS achievement varied across instruments, with the AOFAS score demonstrating the strongest discriminatory capacity, whereas VISA-A and VAS showed more modest PASS performance.

Beyond pain reduction and tendon-specific improvement, restoration of functional gait patterns plays a critical role in patient-perceived recovery in insertional Achilles tendinopathy. Previous rehabilitation research has demonstrated that structured, task-oriented gait rehabilitation can significantly influence perceived usability, confidence, and satisfaction with treatment outcomes, even when underlying pathology persists [46,47]. Ciobanu et al. [46] reported that gait-focused rehabilitation systems improved user satisfaction and functional engagement, highlighting that patients often prioritize global functional performance over isolated symptom metrics.

In this context, the higher PASS achievement observed with the AOFAS score in our study likely reflects both the broader functional scope of the instrument and the intrinsic biomechanical characteristics of insertional Achilles tendinopathy. While the AOFAS score captures global hindfoot function, gait-related activities, and weight-bearing capacity [48], VISA-A and VAS primarily assess tendon-specific symptoms and pain intensity [49]. Given that IAT involves complex enthesis-related biomechanical dysfunction rather than isolated tendon pathology [2,6], improvements in functional gait mechanics and overall foot performance may be perceived as acceptable by patients even in the presence of residual tendon pain.

Consequently, while VISA-A and VAS remain essential tools for monitoring disease-specific pathology and pain severity, the AOFAS score appears to be a more congruent instrument for determining whether a patient’s overall functional state has reached a personally acceptable threshold following intervention.

These findings suggest that in IAT—where pathology involves the enthesis organ complex rather than the tendon substance alone—patient satisfaction depends more strongly on restoration of global hindfoot function than on isolated pain reduction or tendon-specific improvement. VISA-A captured responsiveness to change effectively, but meaningful tendon improvement did not uniformly translate into an acceptable final state. Similarly, pain reduction alone was insufficient in the absence of functional recovery. Collectively, these observations support a multidimensional definition of success in IAT, integrating pain relief, tendon-specific improvement, and overall hindfoot function.

From a clinical decision-making perspective, the predictive values (PPV and NPV) of the identified thresholds further clarify their clinical applicability. The high negative predictive values (NPVs) observed for MCID thresholds—most notably 87.9% for VAS pain reduction—indicate a strong ability to rule out clinically meaningful improvement when score changes fall below the defined cut-offs, thereby facilitating early identification of potential non-responders. In contrast, the substantial positive predictive value (PPV) of the AOFAS PASS threshold (76.1%) provides a quantifiable likelihood that patients achieving this functional score will perceive their condition as acceptable. With respect to radiographic parameters, the moderate PPV (~71%) and NPV (~67%) associated with spur morphology suggest that these measures offer useful prognostic information but should be interpreted in conjunction with clinical assessment rather than as standalone predictors.

Beyond establishing patient-centered outcome benchmarks, this study demonstrates that posterior calcaneal spur morphology significantly modulates the likelihood of achieving PASS after ESWT. While calcaneal spurs have long been recognized as a common radiographic feature of IAT [2,6], prior studies have predominantly treated them as a binary variable (present vs. absent), without assessing their geometric characteristics [15,19,21,23]. By moving beyond this dichotomous classification, our findings advance the paradigm by identifying spur angle and spur length as quantifiable prognostic factors.

Using PASS status as the clinical anchor, ROC analyses identified spur angle ≤ 16° and spur length ≤ 8.7 mm as optimal thresholds discriminating patients who achieved a satisfactory symptom state. Importantly, these parameters were not merely associated with symptom severity but showed direct relevance to patient-defined satisfaction. Clinically, spur length exerted a pronounced effect: nearly 65% of patients with spur length ≤ 8.7 mm achieved PASS, compared with fewer than 40% of those with longer spurs. Spur angulation demonstrated a similar directional trend, with higher PASS rates below the 16° threshold, although this effect was more modest and did not reach conventional statistical significance on univariable comparison.

These findings suggest a hierarchical influence of spur morphology, wherein spur length acts as a dominant mechanical constraint on treatment success, while spur angulation functions as a contributory modifier. This interpretation is reinforced by multivariable analyses, which demonstrated that both increased spur length and increased spur angle independently reduced the odds of achieving MCID and PASS, even after adjustment for demographic factors. Patients with unfavorable morphology exhibited approximately 75–85% lower odds of attaining clinically meaningful improvement or an acceptable symptom state, highlighting the substantial clinical impact of structural anatomy. Importantly, posterior calcaneal spur morphology influenced not only the probability of achieving a patient acceptable symptom state (PASS), but also the likelihood of attaining a minimal clinically important difference (MCID), indicating that unfavorable morphology constrains both the magnitude of improvement and the probability of reaching an acceptable final state.

Importantly, although MCID and PASS are related constructs, they represent conceptually and mechanistically distinct dimensions of treatment success. MCID reflects the magnitude of change perceived as meaningful by the patient and is therefore primarily influenced by biological responsiveness, symptom modulation, and pain reduction. In contrast, PASS represents a final acceptable health state and may be more strongly determined by functional capacity, biomechanical constraints, and the ability to perform daily and gait-related activities. Consequently, predictors of MCID and PASS are not expected to fully overlap.

This distinction is clearly illustrated by our findings on posterior calcaneal spur morphology. While ESWT may effectively modulate pain and inflammatory pathways to yield a clinically meaningful improvement (MCID), persistent mechanical constraints imposed by a prominent spur may limit the maximal functional state a patient can ultimately achieve. As a result, patients may experience meaningful improvement yet fail to reach an acceptable final state when structural or biomechanical limitations persist. Thus, structural anatomy appears to define the ceiling of acceptable function (PASS) rather than the capacity for relative improvement (MCID), reinforcing the need to interpret MCID and PASS as complementary—rather than interchangeable—outcome targets in insertional Achilles tendinopathy.

The clinical relevance of these morphological thresholds is supported by biomechanical and histopathological principles. Increased spur angulation is likely to augment compressive and shear stresses at the Achilles enthesis and retrocalcaneal bursa during dorsiflexion and heel-rise phases of gait, creating a persistent source of mechanical irritation [2,19,22,23]. Similarly, greater spur length enlarges the tendon–bone impingement zone, potentially exceeding the capacity of biologically mediated therapies such as ESWT to induce durable symptom resolution [2,19,23]. Histopathological studies have shown that the insertional region of the Achilles tendon has limited tolerance to sustained compressive loading, rendering it particularly vulnerable to adverse mechanical environments [5].

The graded deterioration observed across the four spur morphology groups further supports a dose–response relationship between increasing structural severity and poorer clinical outcomes. As spur prominence increases, mechanical burden at the enthesis appears to progressively constrain the effectiveness of biologically focused interventions, even when symptomatic improvement is observed at the group level. In this context, unfavorable spur morphology may partially counteract the reparative and neuromodulatory effects stimulated by shockwave therapy, particularly when mechanical overload at the enthesis persists despite biological stimulation [11].

These observations are concordant with established surgical principles, wherein adequate resection of prominent posterosuperior calcaneal bone is regarded as a key determinant of symptom relief in insertional Achilles tendinopathy [2,19]. Importantly, our findings extend this concept into the non-operative domain by introducing objective, pre-treatment radiographic cut-off values that identify patients in whom mechanical factors are likely to limit the effectiveness of biologically driven interventions. These mechanically derived thresholds now allow us to re-examine and explain the long-standing variability in clinical outcomes reported across the ESWT literature.

Our findings contextualize and refine the understanding of ESWT outcomes in IAT established by prior studies, while addressing critical methodological gaps in the existing literature. Regarding treatment efficacy and outcome measurement, systematic reviews of ESWT for IAT report patient satisfaction rates ranging from 64% to 93.8% and statistically significant improvements in VAS, VISA-A, and AOFAS scores at the group level [15,17,40,42,43,50,51]. For instance, Furia et al. [15] reported satisfaction in 73.7% of patients with VAS improving from 7.9 to 2.8 and VISA-A from 47.5 to 76.2. Similarly, Rompe et al. [17] noted 64% satisfaction with VAS decreasing from 7.0 to 3.0. While these studies established ESWT as an effective intervention, they share a fundamental limitation: they rely on unvalidated, non-anchored definitions of “success”—typically using arbitrary thresholds for mean score changes or ordinal satisfaction scales (e.g., “excellent/good” on Roles and Maudsley). Consequently, while demonstrating that ESWT provides some benefit, they cannot determine what constitutes a meaningful benefit for an individual patient or what final state patients consider acceptable.

Our study directly addresses this limitation. By applying anchor-based methodology, we establish that the threshold for a patient-perceived meaningful improvement (MCID) is substantially higher than the average improvement often reported. This explains a critical divergence: despite group-level statistical significance in prior studies (and in our own cohort), only 36.8–43.3% of our patients achieved MCID—a rate markedly lower than the satisfaction percentages commonly cited. For example, Rompe et al. [17] reported 64% satisfaction, whereas only 38.8% of our cohort achieved the MCID for VISA-A. This discrepancy does not negate prior findings but refines them, highlighting that traditional reporting methods may overestimate the proportion of patients who experience truly consequential improvement from their perspective.

Concerning the role of structural pathology, prior studies have yielded inconsistent results regarding calcaneal spurs. Some series, like those by Saxena et al. [50] and Notarnicola et al. [40], reported high satisfaction (82.4% and 93.8%, respectively) without detailed spur analysis. Others, such as Wu et al. [42], observed that patients with concomitant Haglund’s deformity showed less improvement in VISA-A (48.7 to 67.8) compared to those without (49.6 to 83.9), hinting at structural influence. However, most investigations treated spurs as a binary variable (present/absent) [21] and found no significant association with outcomes, creating a paradox given the mechanical rationale for surgery.

Our quantitative morphological analysis resolves this paradox. We demonstrate that specific geometric thresholds—spur angle > 16° and length > 8.7 mm—are prognostically critical, whereas mere presence is not. This explains the heterogeneous outcomes in the literature: cohorts with a higher proportion of patients exceeding these thresholds would report lower satisfaction rates (approaching the ~40% unsatisfactory rate noted by Rompe et al. [17], while those with favorable morphology would achieve higher success. Our work thus reframes “non-responders” not as failures of biological therapy, but as patients whose structural anatomy creates a mechanical environment that constrains ESWT’s effectiveness—consistent with surgical principles where bone resection is fundamental [2,19].

Taken together, these findings build upon and clarify prior evidence by introducing essential methodological rigor (anchor-based MCID/PASS) and anatomical precision (quantitative spur morphology). It provides a coherent framework that explains the spectrum of outcomes in the ESWT literature and offers clinicians practical tools for personalized prognosis and management.

The integration of MCID, PASS, and spur morphology assessment could pave the way for a more evidence-informed approach to treatment strategies, pending validation. Routine lateral ankle radiographs—low-cost and widely available—can be leveraged for prognostic stratification. If prospectively validated, patients with favorable morphology (spur angle ≤ 16°, spur length ≤ 8.7 mm) might be counseled regarding a potentially higher likelihood of achieving a satisfactory outcome with ESWT. Conversely, patients with unfavorable morphology should be informed that, although symptomatic improvement is likely, the probability of reaching an acceptable final state is reduced.

Importantly, this stratification does not imply treatment failure but rather differential probabilities of patient-defined success. In patients with adverse morphology, ESWT may be incorporated into a broader management strategy, including structured loading programs, orthotic modifications, or early discussion of surgical decompression.

Key strengths of this study include a homogeneous IAT cohort, standardized ESWT protocol, rigorous anchor-based outcome methodology, blinded quantitative radiographic analysis, and integration of structural and patient-centered outcomes. Limitations include the retrospective design, which precludes causal inference, and the 6-month follow-up, which reflects intermediate-term outcomes. Longer-term studies are needed to assess durability, particularly in patients with borderline morphology. Additionally, palpation-guided ESWT may introduce targeting variability compared with image-guided techniques, and psychosocial or activity-related factors were not assessed. Furthermore, a key methodological consideration is that the MCID, PASS, and optimal spur morphology thresholds were both derived and evaluated within the same cohort. Although this approach is commonly accepted in exploratory prognostic research, it carries a risk of optimism bias (overfitting), whereby the apparent performance of the thresholds may be inflated for the specific study sample and may not fully generalize to external populations [52,53]. Therefore, the thresholds identified in this study should be interpreted as internally optimized, hypothesis-generating values rather than as definitive, universally applicable cut-off points. Their true clinical accuracy and generalizability must be confirmed through external validation in independent, prospective cohorts.

It is important to emphasize that the prognostic thresholds derived from this study—both for MCID/PASS and for spur morphology—are based on a retrospective, single-center cohort analysis. Although these findings generate robust and novel hypotheses, their generalizability and definitive clinical utility require confirmation through prospective validation in independent, larger cohorts. Accordingly, these thresholds should currently be regarded as investigational tools intended to inform future research and prognostic stratification, rather than as definitive criteria for immediate clinical decision-making. This perspective ensures that the present findings are translated into clinical practice responsibly and guided by higher levels of evidence in the future.

Future research should prospectively validate these morphological thresholds in independent cohorts and explore whether advanced imaging modalities further refine prognostic accuracy. Randomized trials comparing ESWT with early surgical intervention in patients with unfavorable spur morphology would help define optimal treatment pathways.

## 5. Conclusions

This study advances the management of insertional Achilles tendinopathy by moving beyond descriptive radiographic findings to quantitative, prognostically meaningful assessment of posterior calcaneal spur morphology. By establishing treatment-specific MCID and PASS thresholds and identifying spur angle and spur length as key modifiers of patient-defined success, this study proposes a potentially useful, evidence-based framework that, after prospective validation, could aid clinicians in prognostic counseling and personalized treatment selection. Integrating simple radiographic metrics with patient-centered outcome benchmarks represents an important step toward more precise and effective care in IAT.

## Figures and Tables

**Table 1:** Baseline demographic characteristics, clinical scores, and calcaneal spur morphology of the study cohort (*n* = 201).

Parameter	Value
Age (years)	45.08 ± 10.38
Sex, *n* (%)	109 Male (54.2%)/92 Female (45.8%)
Body mass index (kg/m^2^)	26.81 ± 3.52
Baseline VISA-A score	57.77 ± 13.13
Baseline VAS pain score	6.27 ± 1.26
Baseline AOFAS score	61.75 ± 8.39
Spur angle (°)	16.28 ± 6.38 (range: 2.0–32.8)
Spur length (mm)	8.10 ± 2.94 (range: 1.8–16.1)
Symptom duration (months)	(10 [6–16])

Values are presented as mean ± standard deviation unless otherwise stated. Symptom duration is presented as median (interquartile range) due to non-normal distribution. VISA-A, Victorian Institute of Sports Assessment–Achilles; VAS, visual analog scale; AOFAS, American Orthopaedic Foot and Ankle Society Ankle–Hindfoot Score.

**Table 2:** Changes in clinical outcome measures from baseline to 6-month follow-up after ESWT.

Outcome Measure	Baseline	6-Month Follow-Up	Mean Change (Δ)	*p*-Value
VISA-A	57.77 ± 13.13	72.60 ± 14.89	+14.83 ± 8.46	<0.001
VAS pain	6.27 ± 1.26	4.04 ± 1.92	−2.23 ± 1.54	<0.001
AOFAS	61.75 ± 8.39	73.22 ± 11.49	+11.57 ± 8.48	<0.001

Negative change values for VAS indicate pain reduction. *p*-values represent within-group comparisons between baseline and 6-month follow-up.

**Table 3:** Correlation between Global Rating of Change (GROC) scores and change in clinical outcome measures.

Outcome Change Score	Correlation Coefficient (r)	*p*-Value
ΔVISA-A vs. GROC	0.861	<0.001
ΔVAS pain vs. GROC	0.848	<0.001
ΔAOFAS vs. GROC	0.733	<0.001

Correlations were assessed using Spearman’s rank correlation coefficient. Positive r values indicate greater improvement associated with higher GROC scores.

**Table 4:** MCID Thresholds and Responder Rates.

Outcome Measure	MCID Threshold	Patients Achieving MCID, *n* (%)
VISA-A	≥16.5	78 (38.8%)
AOFAS	≥11.5	87 (43.3%)
VAS	≥2.5	74 (36.8%)

MCID thresholds were derived using an anchor-based receiver operating characteristic (ROC) analysis with Global Rating of Change (GROC ≥ 4) as the external criterion. VISA-A, Victorian Institute of Sports Assessment–Achilles; VAS, visual analog scale; AOFAS, American Orthopaedic Foot and Ankle Society Ankle–Hindfoot Score.

**Table 5:** ROC-derived cut-off values and predictive performance metrics for MCID thresholds.

Outcome	Target	AUC (95% CI)	Cut-Off	Sensitivity (%)	Specificity (%)	PPV (%)	NPV (%)
VISA-A	MCID	0.886 (0.841–0.931)	≥16.5 points	77.9	81.2	73.4	84.6
AOFAS	MCID	0.830 (0.767–0.892)	≥11.5 points	76.5	73.7	68.9	79.8
VAS	MCID	0.897 (0.854–0.941)	≤2.5 points	77.9	84.2	74.1	87.9

MCID, minimal clinically important difference; AUC, area under the curve; PPV, positive predictive value; NPV, negative predictive value. Cut-off values were derived using ROC curve analysis based on anchor-defined MCID thresholds.

**Table 6:** PASS Thresholds and Final-State Satisfaction Rates.

Outcome Measure	PASS Threshold	Patients Achieving PASS, *n* (%)
VISA-A	≥70.5	106 (52.7%)
AOFAS	≥72.5	107 (53.2%)
VAS	≤3.5	77 (38.3%)

PASS thresholds represent final absolute scores associated with patient-reported satisfaction. Values are presented as number of patients (percentage). VISA-A, Victorian Institute of Sports Assessment–Achilles; VAS, visual analog scale; AOFAS, American Orthopaedic Foot and Ankle Society Ankle–Hindfoot Score.

**Table 7:** ROC-derived cut-off values and predictive performance metrics for PASS thresholds.

Outcome	Target	AUC (95% CI)	Cut-Off	Sensitivity (%)	Specificity (%)	PPV (%)	NPV (%)
VISA-A	PASS	0.712 (0.641–0.782)	≥70.5 points	70.1	63.5	69.2	64.8
AOFAS	PASS	0.842 (0.787–0.897)	≥72.5 points	80.4	72.1	76.1	77.0
VAS	PASS	0.753 (0.686–0.820)	≤3.5 points	57.7	79.8	65.3	73.6

PASS, patient acceptable symptom state; AUC, area under the curve; PPV, positive predictive value; NPV, negative predictive value. Sensitivity, specificity, PPV, and NPV were calculated at the optimal ROC-derived PASS cut-off values determined using anchor-based methodology.

**Table 8:** ROC-derived cut-off values and predictive performance of posterior calcaneal spur morphology for PASS achievement.

Parameter	Target	AUC (95% CI)	Cut-Off	Sensitivity (%)	Specificity (%)	PPV (%)	NPV (%)
Spur angle	PASS	0.738 (0.668–0.807)	≤16°	69.1	69.2	71.4	66.6
Spur length	PASS	0.713 (0.642–0.785)	≤8.7 mm	75.3	63.5	70.6	68.5

PASS, patient acceptable symptom state; AUC, area under the curve; PPV, positive predictive value; NPV, negative predictive value. Cut-off values were determined using ROC curve analysis with PASS achievement as the outcome and the Youden index for optimal threshold selection

**Table 9:** Association between posterior calcaneal spur morphology and achievement of patient acceptable symptom state (PASS).

**Spur Angle Group**	**PASS Achieved *n* (%)**	**PASS Not Achieved *n* (%)**	***p*** **-Value**
≤16°	59 (59.6%)	40 (40.4%)	
>16°	47 (46.1%)	55 (53.9%)	0.075
**Spur Length Group**	**PASS Achieved *n* (%)**	**PASS Not Achieved *n* (%)**	***p*** **-Value**
≤8.7 mm	72 (64.9%)	39 (35.1%)	
>8.7 mm	34 (37.8%)	56 (62.2%)	<0.001

PASS achievement was defined based on outcome-specific PASS thresholds at 6-month follow-up. *p*-values were calculated using chi-square tests. Spur angle and spur length were dichotomized according to ROC-derived cut-off values.

**Table 10:** Multivariable logistic regression analysis identifying predictors of MCID and PASS achievement after extracorporeal shockwave therapy.

	PASS Threshold		MCID Threshold	
Variable	Adjusted OR (95% CI)	*p*-value	Adjusted OR (95% CI)	
Spur Angle Group				
≤16° (reference)	1.00	—	1.00	—
>16°	0.23 (0.12–0.43)	<0.001	0.16 (0.08–0.32)	<0.001
Spur Length Group				
≤8.7 mm (reference)	1.00	—	1.00	—
>8.7 mm	0.23 (0.12–0.43)	<0.001	0.24 (0.11–0.50)	<0.001
Sex				
Female (reference)	1.00	—	1.00	—
Male	0.91 (0.48–1.74)	0.780	1.14 (0.57–2.29)	0.708
Age (years)	0.996 (0.967–1.027)	0.819	1.02 (0.99–1.06)	0.171
BMI (kg/m^2^)	0.961 (0.878–1.052)	0.389	0.94 (0.85–1.03)	0.174

Adjusted odds ratios (ORs) are presented with 95% confidence intervals (CI). Multivariable models were adjusted for age, sex, and body mass index. Reference categories are indicated. OR values < 1 indicate a reduced likelihood of achieving the corresponding outcome (MCID or PASS), whereas OR values > 1 indicate an increased likelihood of success. MCID, minimal clinically important difference; PASS, patient acceptable symptom state.

## Data Availability

The data presented in this study are available on request from the corresponding author.

## Abbreviations

The following abbreviations are used in this manuscript:

Abbreviation	Definition
IAT	Insertional Achilles Tendinopathy
ESWT	Extracorporeal Shockwave Therapy
MCID	Minimal Clinically Important Difference
PASS	Patient Acceptable Symptom State
VISA-A	Victorian Institute of Sports Assessment–Achilles
AOFAS	American Orthopaedic Foot and Ankle Society
VAS	Visual Analog Scale
ROC	Receiver Operating Characteristic
AUC	Area Under the Curve
GROC	Global Rating of Change
OR	Odds Ratio
ICC	Intraclass Correlation Coefficient
